# Phenolic Composition and Bioactivities of Invasive *Ailanthus altissima* (Mill.) Swingle Leaf Extracts Obtained by Two-Step Sequential Extraction

**DOI:** 10.3390/antiox13070824

**Published:** 2024-07-09

**Authors:** Maria Denisa Cocîrlea, Amalia Soare, Anca Roxana Petrovici, Mihaela Silion, Teodora Călin, Simona Oancea

**Affiliations:** 1 Department of Agricultural Sciences and Food Engineering, “Lucian Blaga” University of Sibiu, 7–9 Ion Ratiu Street, 550024 Sibiu, Romania; denisa.cocirlea@ulbsibiu.ro; 2 National Research and Development Institute for Cryogenic and Isotopic Technologies ICSI, 4 Uzinei Street, 240050 Râmnicu-Vâlcea, Romania; amalia.soare@icsi.ro; 3 Centre of Advanced Research in Bionanoconjugates and Biopolymers, “Petru Poni” Institute of Macromolecular Chemistry, 41A Aleea Grigore Ghica-Voda, 700487 Iasi, Romania; petrovici.anca@icmpp.ro; 4 Physics of Polymers and Polymeric Materials Department, “Petru Poni” Institute of Macromolecular Chemistry, 41A Grigore Ghica Voda Alley, 700487 Iasi, Romania; silion.mihaela@icmpp.ro; 5 Laboratory of Diagnostic and Investigation, Directorate of Public Health, 3 George Barițiu Street, 550178 Sibiu, Romania; teodora.calin65@gmail.com

**Keywords:** *Ailanthus altissima*, polar and non-polar solvents, phenolics, SEM, color changes, antioxidant, antimicrobial

## Abstract

*Ailanthus altissima*, a highly invasive species, contains valuable compounds in different plant parts, indicating great practical potential. This paper proposes the use of non-polar (*n*-hexane) and polar (ethanol) solvents for the extraction of antioxidant compounds from *A. altissima* (family Simaroubaceae) leaves in a sequential two-step process. Fresh and dried leaves were examined for their microstructure by scanning electron microscopy, and for color changes in the CIELAB color space co-ordinates. An investigation of the harvesting season, processing (freezing and drying), and solvent indicates ethanol can be used for the highly efficient extraction of phenolics, flavonoids, tannins, and carotenoids. Statistically significant differences were found between the autumn and summer samples for phenolic content, and between dried and frozen samples for tannin content. The HPLC phenolic profile indicates more phenolics (nine polyphenols) in dried leaves harvested in both seasons compared to those in frozen ones (five to six polyphenols). Frozen leaves showed a higher antioxidant activity in a ferric-reducing antioxidant power assay than that of the dried samples, which exhibited a higher antioxidant activity using the 1, 1-diphenyl-2-picryl-hydrazyl assay, but it was not statistically significant. The phenolic, flavonoid, and carotenoid contents significantly influenced the antioxidant activities. Among the ethanolic extracts, those from dried leaves showed better antibacterial activity, in particular, on *Staphylococcus aureus* and *Enterococcus faecalis*. The high bioactive content and activity of *A. altissima* leaves make them suitable natural raw materials for various applications.

## 1. Introduction

The massive introduction of plant species to biogeographical regions other than their native ones causes a disruption in the structure and function of the ecosystem through invasion. Consequently, these plants, called invasive alien plant species (IAPS), threaten native diversity and cause major economic loss, mainly in the agricultural area [1]. IAPSs have become a serious global concern, so different management strategies have been proposed, including mechanical, biological, or chemical strategies, but not excluding other types based on the valorization of their diverse chemical compounds for purposes such as the production of biopesticides, fibers, bioenergy, or potential pharmaceutical products [2].

*Ailanthus altissima* (Miller) Swingle, also called the Tree of Heaven or China-sumac, is recognized worldwide for its aggressive invasive character [3,4]. Based on the distribution data reported by Sîrbu et al. [5], this species represents one of the most widespread invasive alien plants in the European Union (EU) and is of concern in Romania. The authors present a Romanian map showing the invasiveness of *A. altissima*, where at least one individual was spotted. However, the Tree of Heaven has received significant attention in scientific studies and research due to some historical uses of different parts of the plant in folk medicine (in China and India), such as for the treatment of gastrointestinal diseases [6,7], colds [6], bleedings [8], seborrhoea, and scabies [9]. Several medicinal properties, such as antimicrobial [10,11], neuroprotective [12], anti-inflammatory [9], anti-proliferative [13,14], and DNA-protective [15] properties, have been reported. The antioxidant potential of extracts from different parts of *A. altissima* (bark, leaves, flowers, and fruits) [15,16,17,18,19] has been associated with the species’ increased resistance to pollution [20], features for which this species is frequently cultivated for ornamental purposes [21,22].

The root barks of *A. altissima* are rich in alkaloids, triterpenoids, lignans, coumarins, and chalcone, while the stem barks contain mainly terpenoids (e.g., ailanthone) [23,24,25]. The leaves are rich in polyphenols (tannins and flavonoids) [26,27,28,29] and alkaloids [30,31], while the fruits and seeds contain terpenoids [32], some quassinoid glycosides [33], steroids (e.g., various stigmasterols in the fruits, and ailanthusterol A and B in seeds) [34,35], and phenolics [36]. Polyphenols are the secondary metabolites of all plants and are found in great amounts, in particular, in leaves and fruits, and are among the most abundant compounds, estimated to be at approximately 30% of the world’s total biomass after carbohydrates. Compounds of a polyphenolic structure gain the attribute of being healthy due to their biological properties, in particular, antioxidants [37]. The extraction of antioxidant compounds from the leaves of *A. altissima* has been mainly conducted by Soxhlet, by maceration procedures using a methanol solution at r.t. usually for 24 or 48 h [6,38,39,40] or by decoction at 90–100 °C [28,41].

Natural (poly)phenolic compounds occur either as free molecules or bounded/conjugated, showing polar (hydroxyl groups) and non-polar parts (aromatic ring, quercetin, phenolic terpenes, co-polymers of polyphenols and sugars, phenolic glycosides, phenolics associated with organic acids, amines, and lipids) [42]. Thus, this paper proposes the use of non-polar (*n*-hexane) and polar (ethanol) solvents for the extraction of antioxidant compounds from *A. altissima* leaves in a sequential two-step process. *n*-Hexane is a selective solvent widely used in extraction processes, including those applied in the food (edible oils, fats, flavors, and fragrances) and cosmetic industries [43], due to some physical properties (its high evaporation rate and low boiling point). A maximum residual limit (MRL) of 1 mg/kg was set by the EU for vegetable oils [44], while no regulation for *n*-hexane residue in foods has been indicated by the U.S. Food and Drug Administration (FDA). According to the FDA, *n*-hexane is classified into class 2 (limitation in pharmaceutical products), while the non-toxic eco-friendly solvent ethanol belongs to class 3 (lower risk to human health) [45]. Ethanol, a renewable product that could be obtained by biotechnological processes, is generally recognized as safe (GRAS). To our knowledge, no published studies have been focused on a sequential two-step extraction for the investigated species. The total contents of phenolics, flavonoids, tannins, and carotenoids, as well as the HPLC profile of polyphenols, were investigated in the following types of samples: (1) two-seasonally collected samples from the same geographic area; (2) frozen and dried samples; and (3) polar and non-polar extracts. The antimicrobial activity and total antioxidant activities were also evaluated by FRAP and DPPH assays. In addition to the main objective of the paper, we considered it appropriate to characterize the differently processed and unprocessed raw materials by scanning electron microscopy (SEM) and color attributes, techniques frequently used in association with the compositional analysis of plants [46], in particular, when final products are intended for practical purposes.

## 2. Materials and Methods

### 2.1. Plant Material and Chemical Reagents

**Figure 1:**
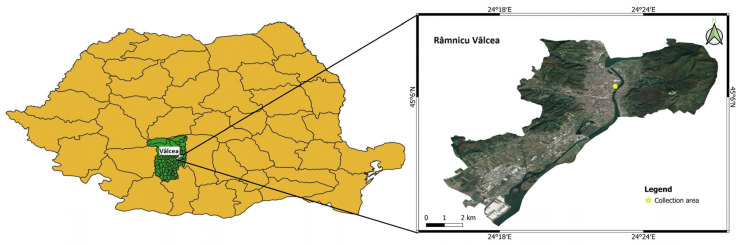
The harvesting area of *A. altissima* leaves: Râmnicu-Vâlcea, Romania. The map was made using the QGIS version 3.28.8 [47].

The leaflets of *A. altissima* (family Simaroubaceae) were collected in summer (June) and autumn (October) from trees and shrubs of different ages originating from the Northern area of the municipality Râmnicu-Vâlcea, Romania (45°6′34″ (N), 24°22′42″ (E)) located at an altitude of 230 m (Figure 1). The voucher specimen of this plant was deposited at the Herbarium (No. HFS 23 1053) of the “Lucian Blaga” University of Sibiu (identification made by lecturer PhD biologist Mihai Crăciunaș, Faculty of Sciences). The young and mature leaves located both towards the base and the top of the individuals were selected. Each of the two groups of leaves (summer and autumn) was divided into two sub-groups according to the type of processing applied (freezing and drying), each one being further subjected to experiments performed in triplicate. One part of the leaf samples from each season was kept in zipper bags at −18 °C and mashed (Blendforce BL 438831) before the extraction process. The other part of the leaf samples from each season was subjected to conventional hot-air drying carried out using the forced-air oven preheated at 50 °C (UFE 400, Memmert, Schwabach, Germany) at a maximum fan speed (100%), until samples reached a final moisture content of about 5–6%, as measured using the moisture analyzer (Mac 210/NP Radwag, Radom, Poland). The dried samples were grounded into powder using the knife mill (Grindomix GM 200, Retsch, Haan, Germany), sieved through standard sieve (pore size 700 μm), and stored at 4 °C until analysis.

Chemical reagents of analytical grade without further purification were used.

### 2.2. Characterization of Fresh and Dried Leaves of A. altissima by SEM and Color Variations

#### 2.2.1. SEM Analysis

The scanning electron microscopy (SEM) technique was used to identify the parts of the fresh leaves and to comparatively evaluate the microstructural characteristics of summer and autumn plant materials, fresh and dried at 50 °C. The SEM analysis was performed using a CZ VP FEG Scanning Electron Microscope (Carl Zeiss, Oberkochen, Germany). In our work, we investigated the backscattered electron (BSE) imaging for dried samples and secondary electron (SE2) imaging for fresh material. The imaging at different magnifications involved an acceleration voltage of 30 kV.

#### 2.2.2. Color Measurements

The color of the acetone extracts of *A. altissima* leaves (fresh and dried at different temperatures, r.t., 30 °C and 50 °C, respectively) was measured using the WinASpect Plus software version 4.0.0.0 (Analytik Jena, Jena, Germany) module of the Specord 200 Plus UV-Vis spectrophotometer (Analytik Jena, Jena, Germany). The color analysis was carried out according to DIN EN ISO 1164 (xyz, CIE L*a*b) and ASTM E 313 (yellowness and whiteness indices) using the standard illuminant D65, field of view 2 and 10, respectively.

The color was expressed as L* (lightness/darkness), a* (red/green), and b* (yellow/blue). The color difference (ΔE), based on the squares of the calculated differences between dried and frozen leaves, was determined according to Equation (1) [48]:

(1) $$\Delta E=\sqrt{{\left(\Delta L*\right)}^{2}+{\left(\Delta a*\right)}^{2}+{\left(\Delta b*\right)}^{2}}$$

### 2.3. Preparation of Polar and Non-Polar Extracts

**Figure 2:**
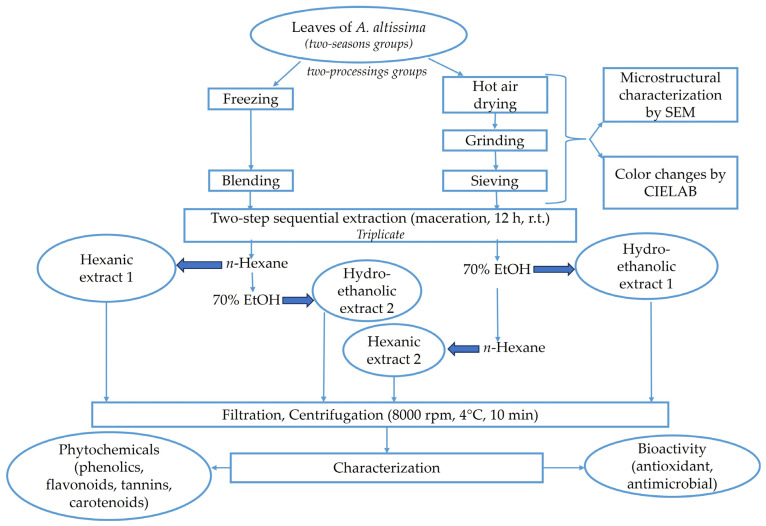
Description of the experimental setup of the two-step sequential extraction for obtaining ethanolic and *n*-hexane extracts from *A. altissima* leaves.

In order to compare the selectivity of extracting polyphenolic compounds from *A. altissima* leaves, frozen and dried, harvested in summer and autumn, a two-step sequential extraction (maceration), using two types of solvent in the order non-polar → polar and polar → non-polar, respectively (Figure 2), was applied under the following conditions:

- Extraction solvents: 70% ethanol was used as polar solvent and *n*-hexane as non-polar solvent;
- Solid/solvent ratio (*w*/*v*): 1/10;
- Extraction temperature by maceration: r.t.;
- Extraction time by maceration: 12 h in dark. Material was soaked in solvent under magnetic stirring (Magnetic stirring rod IKA RO10) at 800 rpm for 3 h, after which the material was stirred occasionally for 3 h, and then left without stirring for 6 h.

The extraction process carried out in triplicate was performed in two steps: at the end of the first extraction, the supernatant (extract 1) was separated from the sediment, and the resulting residue was further extracted (second extraction generating the extract 2) using the solvent of opposite polarity. Before extraction, the residue was left overnight in desiccator and dried in the oven preheated at 35 °C without ventilation until the moisture content reached a value below 10%.

After each extraction, the mixtures were first filtered on Whatman 540 filter paper and, secondly, centrifuged at 8000 rpm, 4 °C for 10 min using the refrigerated centrifuge (Universal 320, Hettich, Berlin, Germany). The extracts (supernatants) were kept at 4 °C until analysis. During the experiments on carotenoids, protection against light and oxygen was assured (no heating was applied as extraction was performed at r.t.).

For the antimicrobial testing, the hydro-ethanolic extracts 1 obtained from maceration at 1/10 (*w*/*v*) solid/solvent ratio were subjected to concentration up to half of their initial volume using a centrifugal vacuum concentrator (RVC 2–18 CD plus, Martin Christ GmbH, Germany).

### 2.4. Content of Phenolics and Carotenoids

The total phenolic content (TPC) was determined spectrophotometrically according to Folin–Ciocalteu method [49]. The Specord 200 Plus UV–Vis spectrophotometer (Analytik Jena, Jena, Germany) was used. The results were expressed in terms of mg gallic acid equivalents (GAEs) per 100 g dry weight (DW).

In the present study, a HPLC-DAD method was performed on hydro-ethanolic extracts prepared from frozen and dried samples collected in both seasons using an Agilent 1260 Infinity Series equipment (Agilent Technologies, Santa Clara, CA USA) with a binary pump and an UV–Vis DAD detector, as previously described by our group [50]. Analysis was carried out under gradient conditions as described, by using 0.1% (*v*/*v*) formic acid in water (mobile phase A) and acetonitrile (mobile phase B), at a flow rate of 0.8 mL/min, using a Mediterranea Sea 18 column (4.6 mm × 150 mm and 5 μm particle size) (Teknokroma Analitica S.A., Barcelona, Spain). The injection volume was 20 µL, the separation process being monitored by a UV–VIS DAD detector at 280 nm. A number of 15 polyphenolic standards was used: gallic acid, protocatechuic acid, vanillic acid, caffeic acid, syringic acid, ferulic acid, *p*-coumaric acid, rosmarinic acid and salicylic acid (as phenolic acids), and rutin, hesperidin, catechin, epicatechin, quercetin, and kaempferol (flavonoids). The stock solutions of standards of 0.5 mg/mL were injected both separately and as a mixture. The mixture was prepared by adding 100 μL of each stock solution, thoroughly homogenized, and injected as such. Chemical reagents and phenolic standards were purchased from Sigma-Aldrich Chemie GmbH (Taufkirchen, Germany).

The total flavonoid content (TFC) was determined spectrophotometrically according to the method described in literature [51]. The results were expressed in terms of mg quercetin equivalents (QEs) per 100 g dry weight (DW).

The total condensed tannin content (TTC) was determined spectrophotometrically according to the method described in literature [52]. The results were expressed in terms of mg catechin equivalents (CEs) per 100 g dry weight (DW).

The content of carotenoids was determined spectrophotometrically according to the method described in literature [53]. The results were expressed in terms of mg β-carotene equivalents per 100 g dry weight (DW).

### 2.5. Antioxidant Activity

#### 2.5.1. Ferric Reducing Antioxidant Power (FRAP)

The total antioxidant activity of the leaf extracts was determined spectrophotometrically using the Ferric Reducing Antioxidant Power (FRAP) assay, according to Benzie and Strain [54]. The results were expressed in terms of mg ascorbic acid equivalents (AAEs) per 100 g dry weight (DW).

#### 2.5.2. Radical Scavenging Activity (RSA) Using the 1, 1-diphenyl-2-picryl-hydrazyl (DPPH) Assay

The RSA of the leaf extracts was determined by the DPPH assay described by Brand-Williams et al. [55]. The extracts were added to a solution of 6 × 10^−5^ M DPPH and the absorbance at 515 nm was measured after 10 min of reaction in darkness at r.t. The results were expressed as inhibition percentage calculated according to Formula (2) [56]:

(2) $$RSA=100\times \frac{A_{0}-A}{A_{0}}$$

where:

*A*_0_ = the absorbance of the DPPH solution (*t* = 0 min);

*A* = the absorbance of the sample in the presence of the DPPH solution (*t* = 10 min).

### 2.6. Antimicrobial Activity

The antimicrobial activity of the crude extracts was tested against standard ATCC (American Type Culture Collection) strains, as follows: Gram-positive (*Staphylococcus aureus* ATCC 25923, *Staphylococcus aureus* clinical isolate, *Streptococcus pyogenes* ATCC 19615, *Enterococcus faecalis* ATCC 29212, and *Bacillus subtilis* ATCC 6633), Gram-negative (*Salmonella enterica* ATCC 13076, *Pseudomonas aeruginosa* ATCC 27853, *Escherichia coli* ATCC 25922, and *Enterobacter aerogenes* ATCC 13084), and fungi (*Candida albicans* ATCC 10231), according to the standardized Kirby–Bauer disk diffusion method [57]. The hydro-ethanolic extracts 1 obtained from the maceration of leaf samples (fresh and dried; collected in the two specified seasons) at 1/10 (*w*/*v*) solid/solvent ratio were subjected to concentration up to half of their initial volume. The concentrated extracts were adsorbed into sterilized paper disks (Macherey-Nagel 615, Germany) of 6 mm diameter, which were further dried at 37 °C. Every disk was loaded with 10 μL extract. The bacteria inoculum was standardized to 0.5 Mc Farland turbidity, corresponding to 1.5 × 10^8^ CFU/mL. The paper disks were added to Müeller–Hinton agar (pH 7.2–7.4) plates and incubated for 20 h at 37 °C. The antimicrobial activity was determined by measuring the diameter (in millimeters) of the inhibition zone around the paper disks. The antifungal activity tested against *Candida albicans* was performed on Müeller–Hinton agar with 2% glucose with methylene blue.

### 2.7. Statistical Analysis

All experimental measurements were performed in triplicate. The results were expressed as “mean value ± standard deviation (SD)”. Statistical analyses were performed using the R 4.3.1 software (available at https://cran.r-project.org/bin/windows/base/, accessed on 30 September 2023) [58]. The differences between groups and the distribution of data on the bioactive composition based on selected criteria (solvent, harvesting period, and processing) were investigated using the function ggboxplot of the ggpubr package, version 0.6.0 [59]. Student’s *t*-test or its non-parametric version, Wilcoxon test, was applied in case the conditions for using the *t*-test were not achieved. Since the data were not normally distributed, the Spearman’s correlation was performed to measure the relationship between pairs of the bioactive compounds’ contents. The correlogram obtained by using the corrplot package version 0.92 [60] was included. The significance of the correlations between the values of analyzed compounds was tested using the rcorr function Hmisc package version 5.1-0 [61]. The statistical modeling by the generalized linear models (GLMs) with gamma distribution and logarithmic link function was used to observe the significance of the influence of bioactive compounds’ content on the antioxidant activities as measured by FRAP and DPPH assays.

## 3. Results and Discussion

### 3.1. Microstructural Properties of Fresh and Dried Leaves by SEM

The microstructural properties of fresh and dried leaves of *A. altissima* have been investigated by SEM analysis. The drying of the leaves was performed at 50 °C in order to remove the moisture (60–70%), which may affect the quality of the final product. The SEM analysis was mainly applied to study the effect of drying and grinding on the surface and morphological properties of the leaves [62].

**Figure 3:**
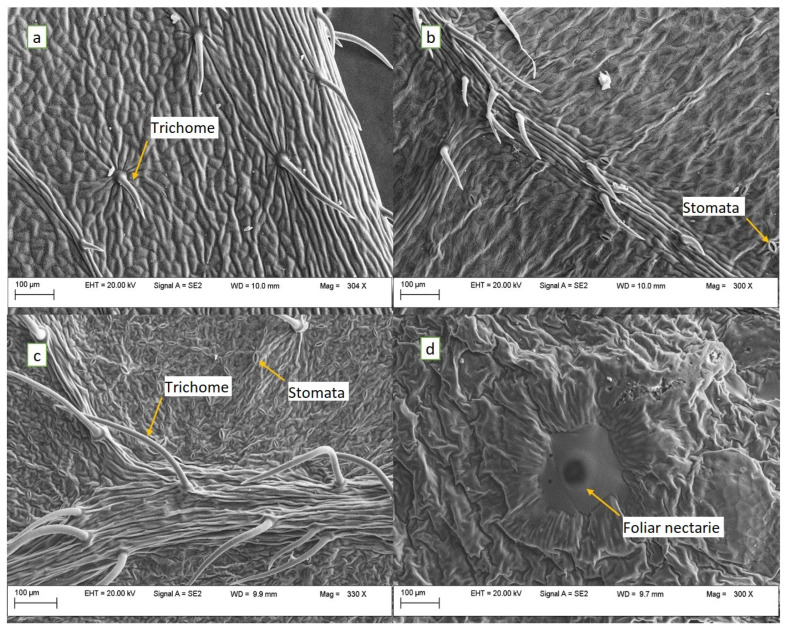
SEM micrographs (300×) of *A. altissima* fresh leaves: (**a**)—trichomes on the upper epidermis; (**b**)—stomata and trichomes on a vein from the upper epidermis; (**c**)—stomata and trichomes on midrib from lower epidermis; and (**d**)—the foliar nectary from the base of lower epidermis.

**Figure 4:**
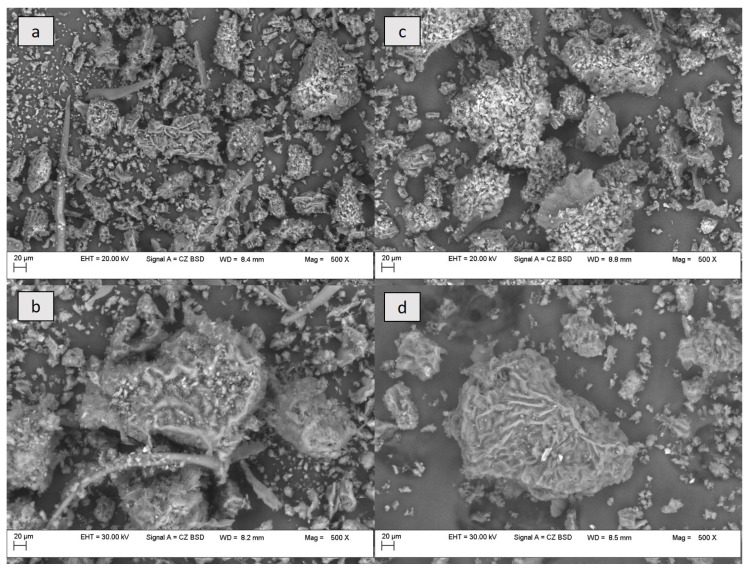
SEM micrographs (500×) of *A. altissima* leaf powder: (**a**,**b**)—summer leaf powder; and (**c**,**d**)—autumn leaf powder. The images include details such as: (**a**,**b**)—trichomes; (**a**)—stomata; and (**b**,**d**)—large fragment of the leaf surface.

The SEM images of fresh and hot-air-dried leaves are shown in Figure 3 and Figure 4. The SEM micrographs of the fresh leaf samples revealed the presence of the trichomes on the epidermal surface, much more representative along the leaf midrib (Figure 3c) and veins (Figure 3b) and less dense on the rest of the leaf surface (upper Figure 3a–c). Additionally, a large number of stomata was found on both the abaxial (Figure 3c) and adaxial (Figure 3b) sides of the leaves. Open nectar-secreting glands (foliar nectaries) were identified at the base of the leaves on the underside (Figure 3d).

The hot-air drying of leaves at 50 °C destroyed the epidermal surface and produced, to some extent, a shrinkage of the glandular trichomes. However, remnants of the trichomes were still identified (Figure 4a,b), but also particles of varying sizes, some of them large enough to show the presence of stomata on their surface (Figure 4a,d). The drying of leaves affected the microstructure of tissues by damaging the cell walls, causing cracks in the samples. Folds oriented in random directions were noticed on the surface of the upper epidermis of the *A. altissima* leaves (Figure 3a), as observed by other authors [63] who explained them as a characteristic of deciduous species. Concerning the presence of epicuticular waxes, Kröber et al. [63] mentioned their presence in an extremely thin layer, which would make it difficult to observe them in ground dried samples and on the surface of the investigated fresh leaves.

### 3.2. Color Changes in Leaf Samples Undergoing Drying

A color assessment using the CIELAB system is often used to study the chemical and quality modifications in food products, food supplements, or other natural products [64]. As color represents a key attribute of a natural product intended for further applications (food, pharmaceutical, or cosmetic), we evaluated how color changes in *A. altissima* leaves could be associated with their processing (heat treatment). A color assessment of samples undergoing drying can help optimize the thermal processing in such a way so as to enhance the color of the final product, which correlates with the preservation of biologically active compounds.

Table 1 shows the changes in the color parameters of leaf extracts obtained from the samples dried at three different temperatures, r.t., 30 °C, and 50 °C, compared to those from the frozen samples, using the CIELAB system (luminosity L*, red-green a*, yellow-blue b*, and color differences ΔE).

The drying of the leaves led to significant color changes irrespective of the applied temperature, mainly due to the chlorophyll degradation caused by heat. The frozen samples presented higher brightness (L*) values than those of the dried ones and were situated in the color range yellow–greenish yellow–green, based on the negative a* and positive b* values. Most of the dried samples with the exception of leaves dried at 30 °C showed positive a* and positive b* values, being situated in the red–orange–yellow range. Drying at 30 °C determined higher L* values and a greenish-green tint of the samples, while drying at 50 °C determined lower L* values and a reddish tint. The samples dried at 30 °C showed a negative a* value (−0.58) and positive b* value (1.80), indicating the residue chlorophyll of the powdered leaves, as shown by other authors [65]. The change in the greenness–redness calculated as a Δa* value between the frozen and the dried leaves was positive, while the difference in yellowness (Δb*) was negative, as a result of the drying process. The reddish tint of the dried samples could result from the presence of anthocyanins synthesized, to some extent, in leaves as potential protectors against solar radiation [66], while the yellow tint detected in all extracts might be due to the presence of flavonoids (quercetin and rutin) and carotenoids [9,15,65]. The overall color difference (ΔE) values, which were situated between 66.00 and 67.68 under the 2° field of view, and between 59.90 and 61.44 under the 10° field of view, indicate significant color changes in the dried samples. Instead, no significant differences in the overall color were found between samples dried at different temperatures. According to Pathare et al. [67], a value of ΔE > 3 is associated with a large difference between the general colors. All investigated extracts presented negative values of the whiteness index, the lowest ones being registered in the frozen leaves; but positive values of the yellowness index, the higher ones being also observed in the frozen samples, which means that the dried samples had a much lower yellow hue and were much more colored (less white) than the frozen ones. To our knowledge, no such investigations on color changes have been performed on *A. altissima* leaves subjected to different processes.

### 3.3. The Influence of Harvesting Season, Processing, and Two-Step Sequential Extraction on the Total Content of Bioactive Compounds in A. altissima Leaves

The recovery of bioactive compounds from *A. altissima* leaves was proposed to be conducted using a two-step sequential extraction (Figure 2), in two ways—(a) starting with a polar protic solvent (hydro-ethanolic solution) and progressing in the second step with a non-polar solvent (*n*-hexane); and (b) starting with a non-polar solvent (*n*-hexane) and progressing in the second step with a polar solvent (hydro-ethanolic solution)—to provide the extraction of a broad range of compounds with different polarities.

Higher amounts of the investigated compounds were obtained using the sequential two-step extraction starting from a non-polar to polar solvent, in the case of the dried samples, while the opposite was obtained in the case of frozen samples, with the extraction from polar to non-polar giving better results. However, in all experiments, under the tested conditions, ethanol was the solvent that efficiently extracted bioactive compounds from dried and frozen samples harvested either in autumn or summer. Highly significant differences were found between the ethanol and *n*-hexane in extracts obtained either from the starting material (first extraction in ethanol; extract 1) or from the residue subjected to the second extraction in ethanol (extract 2) (Figure 2). Low amounts of the investigated compounds were still extracted into *n*-hexane, part of them (tannins and carotenoids) being better isolated from the dried leaves, while others (flavonoids) were better isolated from the frozen ones.

The highest content of the investigated bioactive compounds was obtained as follows: TPC (7256.92 ± 13.65 mg GAE/100 g DW) in ethanolic extract (polar to non-polar sequential extraction) from frozen leaves harvested in autumn; TFC (9197.65 ± 53.43 mg QE/100 g DW) in ethanolic extract (polar to non-polar sequential extraction) from frozen leaves harvested in summer; TTC (990.35 ± 0.48 mg CE/100 g DW) in ethanolic extract (both types of sequential extraction) from dried leaves harvested in summer; and carotenoid content (108.18 ± 0.64 mg β-carotene/100 g DW) in ethanolic extract (both types of sequential extraction) from dried leaves harvested in summer. The lowest contents of the investigated bioactive compounds were obtained as follows: TPC (3791.43 ± 4.76 mg GAE/100 g DW) in ethanolic extract (non-polar to polar sequential extraction) from frozen leaves harvested in summer; TFC (6020.88 ± 35.90 mg QE/100 g DW) in ethanolic extract (non-polar to polar sequential extraction) from frozen leaves harvested in autumn; TTC (233.68 ± 3.39 mg CE/100 g DW) in ethanolic extract (non-polar to polar sequential extraction) from frozen leaves harvested in summer; and carotenoid content (55.60 ± 0.11 mg β-carotene/100 g DW) in ethanolic extract (polar to non-polar sequential extraction) from dried leaves harvested in autumn.

**Figure 5:**
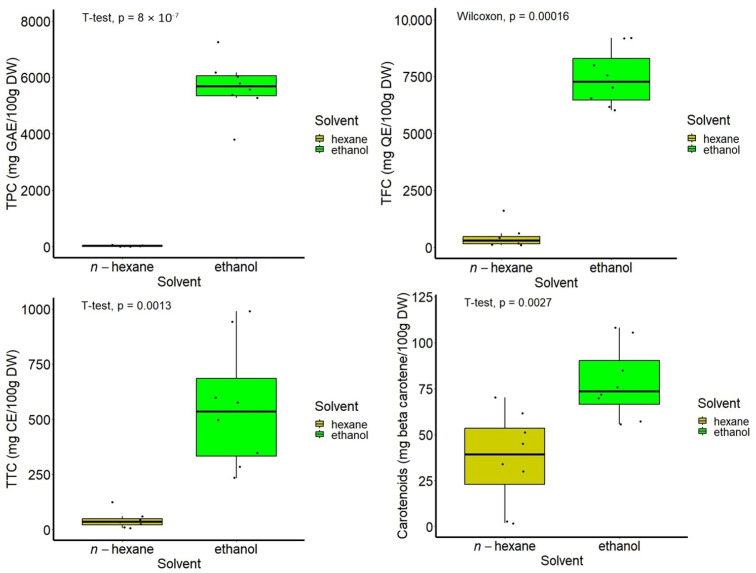
Boxplot representation of the content of bioactive compounds, according to the type of solvent used in the two-step sequential extraction.

Figure 5 illustrates the boxplot representation of data on the composition of all types of samples, showing the values of the compounds’ content distributed according to the solvent type, with the differences being highly significant.

**Figure 6:**
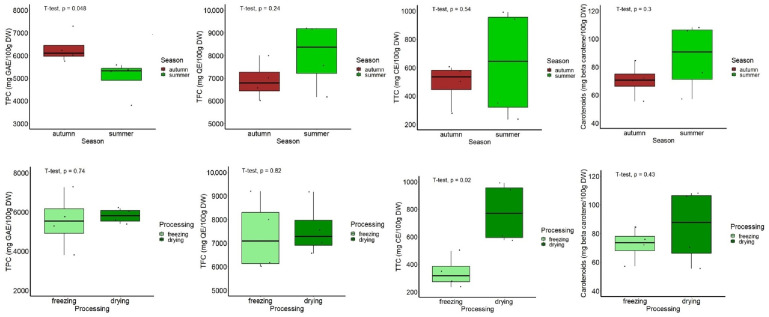
Boxplot representation of the content of bioactive compounds in hydro-ethanolic extracts, according to the harvest period and leaf processing.

Among the hydro-ethanolic extracts, those obtained from leaves harvested in autumn showed a significantly higher TPC than that of samples harvested in summer (*p* < 0.05), while an opposite trend was found in the case of TFC, TTC, and carotenoid content, but with no statistical significance between the two seasons (*p* > 0.05), as indicated in Figure 6. With respect to the sample processing, the hydro-ethanolic extracts obtained from the dried leaves showed a significantly higher TTC (*p* < 0.05), with similar trends being observed for the content of the other compounds, but they were not statistically significant (*p* < 0.05, Figure 6).

Summarizing the data on the efficiency of the extraction of polyphenolic compounds (TPC, TFC, and TTC) according to the proposed sequential procedure (Figure 2), the pre-processing (freezing and drying) mainly influenced the content in all investigated samples. The order of solvent fractions obtained from frozen leaves harvested in either of the both seasons was as follows: hydro-ethanolic extract 1 > hydro-ethanolic extract 2 > hexanic extract 1/hexanic extract 2. In the case of the dried leaves, polyphenolic compounds were efficiently extracted in the order of hydro-ethanolic extract 2 > hydro-ethanolic extract 1 > hexanic extract 1/hexanic extract 2. Thus, the direct extraction of compounds in ethanol is recommended for frozen leaves, while the second extraction in ethanol from the residue that remained after the first extraction in *n*-hexane was efficient in the case of the dried leaves.

**Figure 7:**
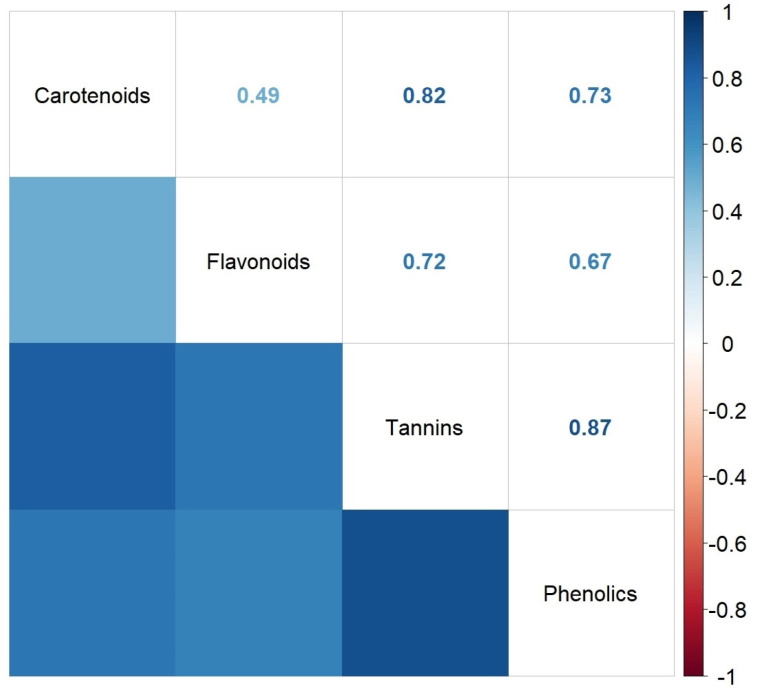
Correlation plot (correlogram) of the Spearman’s correlation coefficients between contents of investigated bioactive compounds.

In order to determine the degree of association between the contents of various bioactive compounds for the particular leaf extract of *A. altissima*, the regression analysis was performed for these independent variables. The correlation plot (correlogram) of the Spearman’s correlation coefficients between pairs of content values of the investigated bioactive compounds of *A. altissima* leaf extracts is presented in Figure 7.

As shown in Figure 7, the correlations between the investigated phytochemicals were positive and highly significant at *p* < 0.05, with the exception of the correlation between flavonoids and carotenoids, which was marginally significant (*p* = 0.0517). The strongest positive correlations were found between phenolics or carotenoids and tannins (r = 0.87 and r = 0.82, respectively). A significant positive correlation between phenolics and tannins was also found by other authors on chestnut leaves (*p* < 0.01 and r = 0.64), but, unlike our study, they found a negative correlation (not significant) between flavonoids and tannins (r = −0.34) [68]. Other published results, e.g., on ginger rhizome, reported a significant positive correlation between phenolics and carotenoids (r = 0.915) similar to our study (r = 0.73) [69]. A study published on bioactive compounds from raspberries showed that flavonoids correlated negatively with carotenoids (−0.738) [70]. According to these results, the correlation significance of each phytochemical may vary with plant origin.

The HPLC-DAD analysis of the phenolic profile of the investigated four types of leaf hydro-ethanolic extracts was performed by using 15 reference standards, which were analyzed both one by one and in a mixture of equal proportions. The retention times (RTs) are listed in Table 2. The identification of the chromatographic peaks in samples was accomplished by comparing their RTs with those of the known commercial standards and with the UV spectra at 280 nm by using a diode array detector.

**Figure 8:**
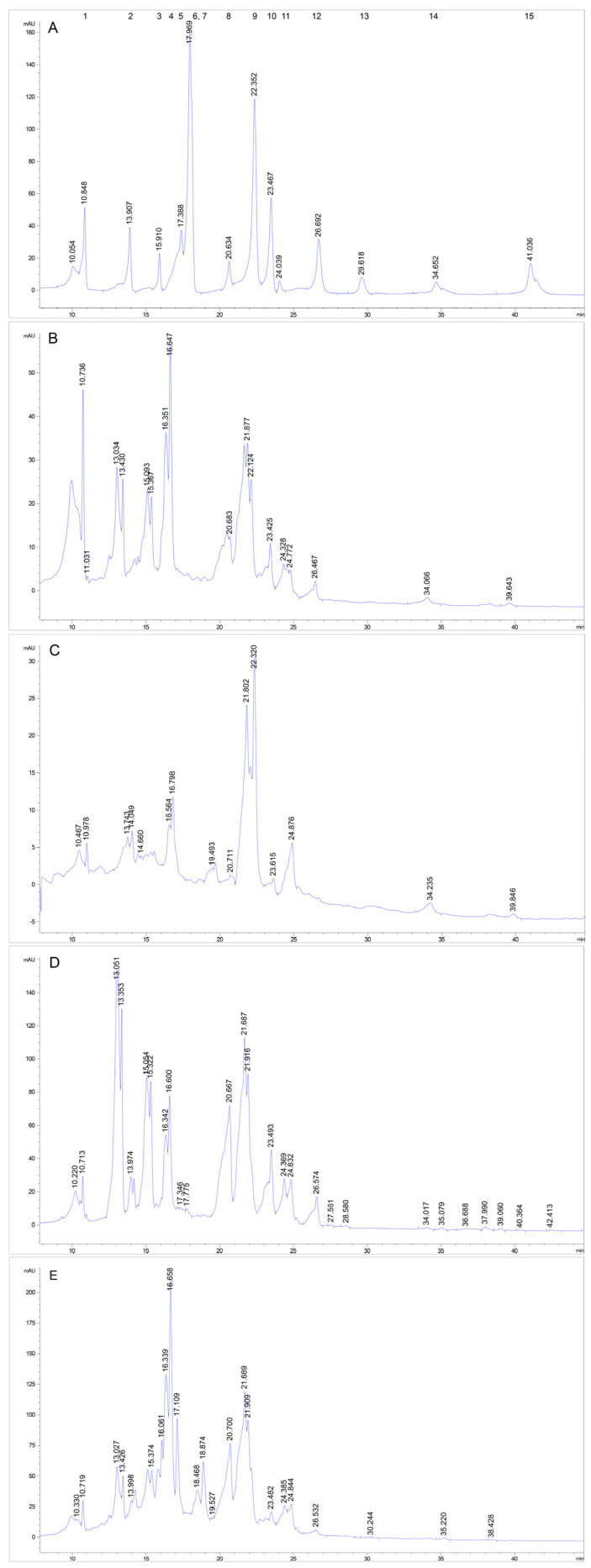
HPLC comparative chromatograms of (**A**)—standards: **1** gallic acid, **2** protocatechuic acid, **3** catechin, **4** vanillic acid, **5** epicatechin, **6** caffeic acid, **7** syringic acid, **8** rutin, **9** ferulic acid, **10** *p*-coumaric acid, **11** hesperidin, **12** rosmarinic acid, **13** salicylic acid, **14** quercetin, and **15** kaempferol; (**B**) crude extract from summer frozen sample; (**C**) crude extract from autumn frozen sample; (**D**) crude extract from summer dried sample; and (**E**) crude extract from autumn dried sample.

As noticed in the chromatogram (Figure 8A), due to the inter-molecular interactions between all 15 polyphenolic compounds in solution, a slight shift of the RTs occurred and the peaks of four polyphenols overlapped two by two in the mixture chromatogram. However, in the chromatograms of the hydro-ethanolic leaf extracts (Figure 8B–E), the separation is evident for epicatechin and vanillic acid.

The HPLC-DAD phenolic profiles of the investigated leaf extracts by using reference standards revealed great differences, in particular, between frozen and dried leaf extracts. A number of nine polyphenolic compounds (gallic acid, protocatechuic acid, catechin/epicatechin, vanillic acid, rutin, *p*-coumaric acid, hesperidin, rosmarinic acid, and quercetin) was identified in dried leaves harvested in both seasons, while fewer compounds were found in frozen leaves, as follows: five polyphenols (gallic acid, protocatechuic acid, vanillic acid, ferulic acid, and *p*-coumaric acid) in frozen leaves harvested in autumn and seven polyphenols (gallic acid, vanillic acid, rutin, ferulic acid, *p*-coumaric acid, and hesperidin) in frozen leaves harvested in summer, respectively. Ferulic acid was identified only in the frozen samples, while quercetin only in the dried samples. By comparing the results on the frozen samples in relation to the season, it was found that rosmarinic acid, rutin, and hesperidin occur only in summer leaves, while protocatechuic acid occurs only in autumn leaves. Within the dried samples in relation to the season, the only difference was that catechin was detected in autumn leaves, while epicatechin was detected in summer leaves. Caffeic acid, syringic acid, salicylic acid, and kaempferol were not identified in the four investigated extracts.

To our knowledge, no studies on the variation of the content of bioactive compounds from *A. altissima* leaves with the pre-treatment (freezing and drying) and season (summer and autumn) have been identified so far. The determination of the proper harvesting season of a plant material is crucial for obtaining products rich in valuable chemical compounds [71,72]. The extract obtained from *A. altissima* autumn dried leaves, rich in polyphenols, was studied by our group also in relation to its eco- and cyto-toxic effects on target and non-target organisms, with the results on such effects and polyphenolic profile being reported [50].

The study of Marinaș et al. [73] on different invasive plants, including *A. altissima,* from the Romanian Botanical Garden of Bucharest, showed the presence of seven polyphenols in extracts prepared in almost similar conditions as ours with slight differences (drying at room temperature, and extraction in ultrasonic bath), polyphenolic compounds which are common to the ones hereby identified—gallic acid, protocatechuic acid, catechin, epicatechin, rutin, *p*-coumaric acid, and quercetin—but they also found low amounts of caffeic acid, syringic acid, and ferulic acid and other polyphenols (4-hydroxybenzoic acid, chlorogenic acid, ellagic acid, and resveratrol). Similar to the results of Andonova et al. [15] on hydro-ethanolic extracts but obtained under reflux at 70 °C from dried *A. altissima* leaves of Bulgaria, our investigation showed the presence of five common flavonoids in the extracts obtained from dried leaves: catechin, epicatechin, rutin, hesperidin, and quercetin. Despite the fact that gallic acid and quercetin were also reported in most studies [15,27,73], Luís et al. [6] did not detect them in a leaf extract obtained by Soxhlet extraction with a water/ethanol mixture (50/50; *v*/*v*) from *A. altissima* leaves of Portugal, which were previously dried at 35 °C. The differences in the phenolic profile can be explained by the variations in the harvest period (here, summer and autumn samples were investigated), which highly impact the phenolics biosynthesis in plants, the plant origin (different geographical areas), the processing/pre-treatment (mechanical, freezing, drying, and grinding), and the extraction conditions, in particular, the solvent type (effect on the membrane disruption and compound release) and the temperature, all of which influence, to a certain extent, the isolation of a particular phenolic compound.

### 3.4. Antioxidant Activity

The in vitro total antioxidant activity of the leaf extracts was determined using an electron-transfer-based assay (FRAP) and a stable free-radical-based assay (DPPH), methods frequently applied to natural products.

The hydro-ethanolic extracts showed a higher antioxidant activity by FRAP within the range of values 5043.59–8522.58 mg AAE/100 g DW depending on the sample type than that of the *n*-hexane extracts (0.79–79.95 mg AAE/100 g DW). Increased values were obtained in those hydro-ethanolic extracts prepared from frozen leaves (summer and autumn). Similarly, the hydro-ethanolic extracts proved to be the strongest DPPH scavengers, showing values between 79.11% and 94.50% depending on the sample type, while the *n*-hexane extracts showed values between 1.85% and 44.45%. The extract obtained from frozen autumn leaves using the non-polar to polar sequential process indicated the highest antioxidant activity by FRAP (8522.58 ± 73.86 mg AAE/100 g DW). Instead, the extract obtained from dried summer leaves by both types of sequential processes indicated the highest antioxidant activity by DPPH (94.50 ± 0.02%).

Other reported values of the antioxidant activity of dried *A. altissima* leaves by the FRAP assay, expressed as Trolox equivalents (TEs) or ascorbic acid equivalents (AAEs), are as follows: 906.01 mmol TE/g DW for hydro-ethanolic extracts [15], 242.5–334 μmol TE/g for methanolic extracts [27], and 88.7 μmol AAE/g for methanolic extracts [74]. The literature reports DPPH values of 88.5% for a methanolic extract of dried *A. altissima* leaves and 7.3% for a *n*-hexane extract, respectively [74].

**Figure 9:**
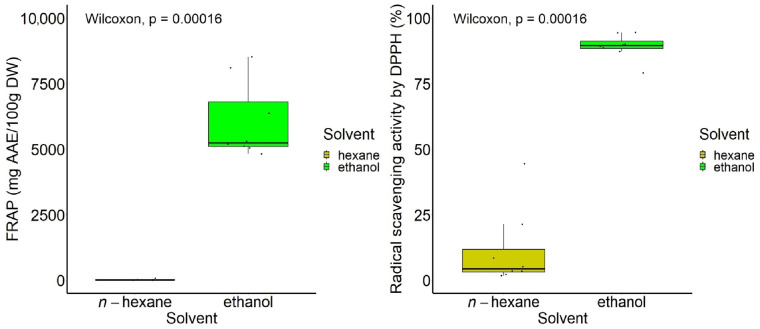
Boxplot representation of the total antioxidant activity (FRAP and DPPH) according to the type of solvent.

Ethanol provided a high recovery of antioxidant compounds expressed in total antioxidant activity for all experiments, under the tested conditions, as shown in Figure 9. Highly significant differences in antioxidant activities were found between ethanol and *n*-hexane in extracts obtained either through *n*-hexane → ethanol sequential process or ethanol → *n*-hexane sequential extraction.

**Figure 10:**
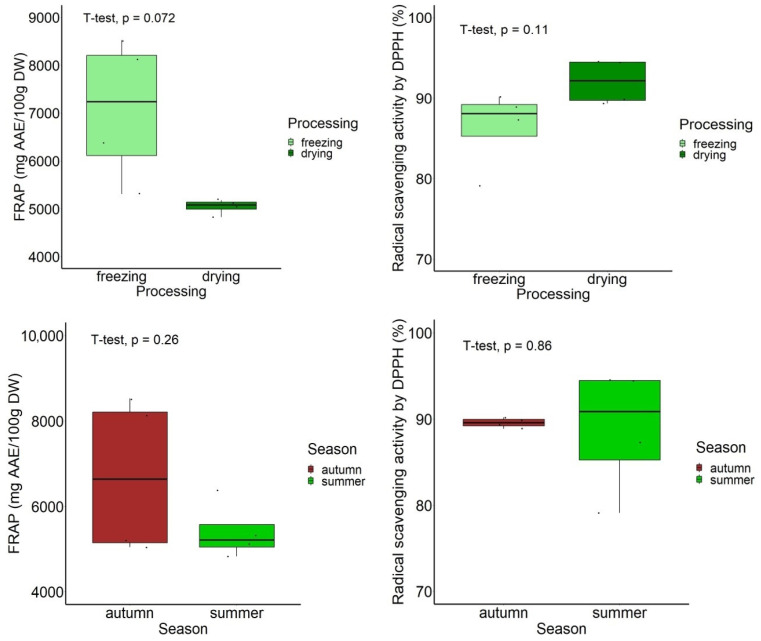
Boxplot representation of the total antioxidant activity (FRAP and DPPH) in hydro-ethanolic extracts according to the leaf processing and harvesting season.

According to the leaf processing by freezing or drying at 50 °C, no significant differences were found (*p* > 0.05), despite the fact that the FRAP values were higher in the frozen samples (*p* = 0.072; marginally significant), while the DPPH values were greater in the dried samples (*p* = 0.110) (Figure 9). According to the harvesting season, no significant differences (*p* > 0.05) were found between the autumn and summer samples, in relation to the antioxidant activity (Figure 10).

A significant positive correlation was found between the FRAP and DPPH values (*p* = 0.0006, r = 0.76). A strong positive association between the two antioxidant assays was also found by other authors [75].

Summarizing the data on the antioxidant activity of leaf extracts as measured by the FRAP assay, the season (summer and autumn) dictated the order of the solvent fractions according to the proposed sequential procedure (Figure 2), as follows: hydro-ethanolic extract 1 > hydro-ethanolic extract 2 > hexanic extract 1 > hexanic extract 2 for summer leaves, and hydro-ethanolic extract 2 > hydro-ethanolic extract 1 > hexanic extract 1 > hexanic extract 2 for autumn leaves. A direct extraction of antioxidant compounds in ethanol is recommended for summer leaves, while a second extraction in ethanol from the residue that remained after the first extraction in *n*-hexane was efficient in autumn leaves.

In order to identify the significant compound predicting the antioxidant activities as measured by FRAP and DPPH in *A. altissima* leaf extracts in relation to harvesting seasons, sample processing, and extraction conditions, the statistical GLM modeling analysis was applied.

In samples harvested in summer, flavonoids were found to be the significant predictor for the antioxidant activity by FRAP (GLM, *p* = 0.000125, t = 8.730), while total phenolics better predicted the antioxidant activity by DPPH (GLM, *p* = 0.000565, t = 6.636). In samples harvested in autumn, total phenolics were the best predictor for the antioxidant activity by FRAP (GLM, *p* = 0.000106, t = 8.983), while carotenoids better predicted the antioxidant activity by DPPH (GLM, *p* = 0.00093, t = 6.042).

In samples subjected to freezing, according to the GLM analysis, the predictor that had a significant influence on the antioxidant activities (FRAP and DPPH) was found to be the flavonoids (GLM, *p* = 0.0000472, t = 10.363 for FRAP; and *p* = 0.000323, t = 7.358 for DPPH, respectively). Instead, in dried samples, total phenolics significantly predicted the antioxidant activities (FRAP and DPPH) (GLM, *p* = 0.000211, t = 7.950 for FRAP; and *p* = 0.016453, t = 3.298 for DPPH, respectively).

In relation to the extraction process, the GLM modeling analysis showed that the first step generated extracts in which total phenolics were found to be the significant predictor for the antioxidant activity by FRAP (GLM, *p* = 0.000140, t = 8.555), while carotenoids were found to be significant predictors for the antioxidant activity by DPPH (GLM, *p* = 0.00411, t = 4.499). The second step of extraction (from residues that remained from the first extraction) led to extracts in which flavonoids were found to be the significant predictors for the antioxidant activity by FRAP (GLM, *p* < 0.001, t = 9.414), while total phenolics significantly predicted the antioxidant activity by DPPH (GLM, *p* = 0.00678, t = 4.043).

Our results are in agreement with other ones reporting that the significant antioxidant activity in plants could be mainly attributed to phenolics and flavonoids. Positive associations between flavonoids or total phenolics and the antioxidant activities by FRAP or DPPH of *A. altissima* leaves, according to correlation studies, have been reported by other authors, without investigating them in relation to environmental or extraction factors [16]. There are few studies on the correlation between carotenoids and antioxidant activities, but a negative correlation (based on a linear regression analysis) was found between carotenoids and DPPH in leaf extracts of *Phyllanthus emblica*, while a positive correlation between carotenoids and DPPH was found in the fruit extract of the same species [76]. The absence of any association between tannins and antioxidant activity (DPPH) has been also reported in other leaf extracts [77].

### 3.5. Antimicrobial Activity

The antimicrobial activity of four hydro-ethanolic crude extracts of *A. altissima* leaves (frozen and dried, and harvested in summer and autumn) was investigated against nine pathogenic bacterial strains and one fungal strain. The results reported in terms of the size of the zones of inhibition of bacterial growth are presented in Table 3.

The antibacterial activity of several standard antibiotics against Gram-positive and Gram-negative strains according to EUCAST 2023 [78] and CLSI 2022(M100) [79] is given in Table 4 with the purpose of carrying out a comparison with the inhibition zones of the samples.

Among the ten bacterial strains hereby investigated, five Gram-positive and one Gram-negative bacteria showed sensitivity to at least one of the crude leaf extracts, in particular, those obtained from dried leaves. While *Streptococcus pyogenes* and *Bacillus subtilis* strains were slowly inhibited only by the hydro-ethanolic extract of dried leaves harvested in autumn (with a growth inhibition zone of 8 mm), the *Staphylococcus aureus* and *Enterococcus faecalis* strains were sensitive to all type of extracts, showing a high sensitivity to extracts from dried samples with inhibition zones of 10 mm comparable to those of the standard antibiotic vancomycin, which showed an inhibition zone of 10–16 mm against *Enterococcus faecalis*. Among the tested Gram-negative bacteria, *Escherichia coli* showed some sensitivity to the dried leaf extract from autumn, as well as from frozen summer leaves.

Among the investigated extracts, those obtained from autumn leaves, which were dried at 50 °C, indicated better antibacterial activity. When compared to standard synthetic antibiotics, the inhibition zone diameters of the natural extracts are lower in most cases. No antifungal activity of the investigated extracts has been found on *Candida albicans* ATCC 10231 strain. Albouchi et al. [27] reported somewhat similar results but using concentrated methanolic extracts from *A. altissima* dried leaves, which were effective against Gram-positive bacteria (*Staphylococcus aureus, Enterococcus feacium, Streptococcus agalactia,* and *Bacillus subtilis*) with inhibition zones ranging from 10 to 24 mm in relation to the strains and geographical location of samples collected from three different Tunisian regions; and some Gram-negative bacteria, e.g., *Escherichia coli* with an inhibition zone of 10 mm, while no activity has been shown on *Salmonella typhimurium* and *Candida albicans*. Instead, the antibacterial activity of a methanolic leaf extract against the strain *Pseudomonas aeruginosa* KCTC 2004 and no antibacterial activity on the strain *Escherichia coli* ATCC 43888 were reported by Caramelo et al. [9]. The differences in the results reporting the antimicrobial activity of the extracts may be linked to variations in the origin of the *A. altissima* samples, climatic conditions of growth, harvesting season, and processing conditions (pre-treatments, extraction procedures, concentration of extracts, and type of microbial strains). However, the obtained results confirm the historical use of this species in folk medicine, for example, in wound healing [24].

## 4. Conclusions

The invasive species *A. altissima* represents a cheap and affordable source of bioactive compounds of industrial interest. However, the chemical composition of leaves varies with the season and processing of raw materials.

This paper reports, for the first time, a two-step sequential process using non-polar (*n*-hexane) and polar (ethanol) solvents for the extraction of antioxidant compounds from *A. altissima* leaves, either frozen or dried, and harvested in summer or autumn. The results showed significant differences between the two solvents in favor of 70% ethanol, which efficiently extracted phenolics, flavonoids, tannins, and carotenoids, despite the fact that low amounts of these bioactive compounds were also recovered from the *n*-hexane fractions. Statistically significant differences were found between autumn and summer samples for phenolic content and between dried and frozen samples for tannin content. The HPLC phenolic profile indicates more phenolics (nine polyphenols, of which there were five phenolic acids and four flavonoids) in the dried leaves harvested in both seasons compared to those of the frozen samples (five to six polyphenols).

Processing and seasonal variation showed that frozen autumn leaves exhibited higher antioxidant activity by FRAP, while dried summer leaves exhibited slightly greater radical scavenging activity (DPPH), but it was not statistically significant. Among the investigated samples, the dried leaves showed better antibacterial activity, in particular, on *Staphylococcus aureus* and *Enterococcus faecalis* strains.

The results hereby obtained indicate the optimal harvesting, processing, and extraction conditions for a high recovery of antioxidant compounds from a renewable biomass and give scientific support for the traditional medicinal use of this invasive species. From a practical point of view, the results reporting a high content of phenolics encourage the use of the leaves for purposes such as antioxidant or antimicrobial preservatives for different industries that convert these raw materials into functional ingredients.

## Figures and Tables

**Table 1:** CIELAB parameters of frozen and dried *A. altissima* leaves.

Sample	Material Processing	L*	a*	b*	ΔE	Whiteness Index	Yellowness Index
*A. altissima* leaf acetone extract	2° field of view						
	Freezing	27.68 ± 0.82	−43.90 ± 0.05	44.01 ± 0.07	-	−14.81	96.01
	Drying (r.t.)	0.65 ± 0.11	0.46 ± 0.04	0.83 ± 0.16	67.54	−0.14	74.19
	Drying (30 °C)	1.26 ± 0.05	−0.58 ± 0.07	1.80 ± 0.04	66.00	−0.32	83.86
	Drying (50 °C)	0.38 ± 0.05	0.09 ± 0.04	0.41 ± 0.05	67.68	−0.06	63.04
	10° field of view						
	Freezing	26.63 ± 2.66	−35.05 ± 1.46	43.32 ± 2.83	-	−14.81	96.01
	Drying (r.t.)	0.60 ± 0.10	0.42 ± 0.06	0.73 ± 0.13	61.23	−0.14	74.19
	Drying (30 °C)	1.16 ± 0.28	−0.40 ± 0.05	1.63 ± 0.42	59.90	−0.32	83.86
	Drying (50 °C)	0.36 ± 0.17	0.15 ± 0.06	0.36 ± 0.07	61.44	−0.06	63.04

**Table 2:** Retention time (RT) of polyphenolic compounds recorded in the individual polyphenol injections, in the polyphenol mixture, and in the hydro-ethanolic leaf extracts of *A. altissima*.

	Sample	Polyphenols as Individual Run	Polyphenols in Mixture Run	Frozen (Summer)	Frozen (Autumn)	Dried (Summer)	Dried (Autumn)
Polyphenols		RT (min)					
Gallic acid		10.97	10.84	10.73	10.97	10.71	10.71
Protocatechuic acid		13.98	13.90	-	14.04	13.97	13.99
Catechin		15.63	15.91	-	-	-	15.79
Vanillic acid		16.74	overlapped with epicatechin	16.64	16.79	16.6	16.65
Epicatechin		17.28	17.38	-	-	17.34	-
Caffeic acid		17.49	17.96	-	-	-	-
Syringic acid		18.09	overlapped with caffeic acid	-	-	-	-
Rutin		20.42	20.63	20.68	-	20.66	20.70
Ferulic acid		22.38	22.35	22.12	22.32	-	-
*p*-Coumaric acid		23.10	23.46	23.42	23.61	23.49	23.48
Hesperidin		24.01	24.03	24.32	-	24.36	24.38
Rosmarinic acid		26.33	26.69	26.46	-	26.57	26.53
Salicylic acid		29.31	29.61	-	-	-	-
Quercetin		34.72	34.65	-	-	35.07	35.22
Kaempferol		39.71	41.00	-	-	-	-

“-” = not detected.

**Table 3:** Inhibition zones (mm) of bacterial growth in the presence of different hydro-ethanolic extracts of *A. altissima* leaves.

	Sample	Zone of Inhibition (mm)			
Microbial Strain		Frozen (Summer)	Frozen (Autumn)	Dried (Summer)	Dried (Autumn)
*Staphylococcus aureus* ATCC 25923		-	-	8.0 ± 0.1	8.0 ± 0.1
*Staphylococcus aureus* clinical isolate		8.0 ± 0.1	8.0 ± 0.1	10.0 ± 0.2	10.0 ± 0.2
*Streptococcus pyogenes* ATCC 19615		-	-	-	8.0 ± 0.1
*Enterococcus faecalis* ATCC 29212		8.0 ± 0.1	9.0 ± 0.2	9.0 ± 0.2	10.0 ± 0.3
*Bacillus subtilis* ATCC 6633		-	-	-	8.0 ± 0.1
*Salmonella enterica* ATCC 13076		-	-	-	-
*Pseudomonas aeruginosa* ATCC 27853		-	-	-	-
*Escherichia coli* ATCC 25922		9.0 ± 0.2	-	-	10.0 ± 0.2
*Enterobacter aerogenes* ATCC13084		-	-	-	-
*Candida albicans* ATCC 10231		-	-	-	-

“-” = no inhibition.

**Table 4:** Antibacterial activity of several known standard antibiotics against bacterial strains of interest.

Reference Strain	Antibiotic	Zone of Inhibition (mm)
*Staphylococcus aureus* ATCC 25923	Gentamicin 10 μg/disc	19–27
	Vancomycin 30 μg/disc	17–21
*Streptococcus* spp. Beta-haemolitic group	Penicillin 10 μg/disc	≥24
	Vancomycin 30 μg/disc	≥17
*Enterococcus faecalis* ATCC 29212	Gentamicin 30 μg/disc	12–18
	Vancomycin 5 μg/disc	10–16
*Pseudomonas aeruginosa* ATCC 27853	Ciprofloxacin 5 μg/disc	25–33
	Gentamicin 10 μg/disc	17–23
*Escherichia coli* ATCC 25922	Ampicillin 10 μg/disc	15–22
	Gentamicin 10 μg/disc	19–26

## Data Availability

All of the data are contained within the article.
